# Progranulin mediates the onset of pristane induced systemic lupus erythematosus

**DOI:** 10.1186/s42358-024-00405-8

**Published:** 2024-09-09

**Authors:** Michun He, Aubryanna Hettinghouse, Yufei Bi, Yuehong Chen, Chuanju Liu

**Affiliations:** 1 Department of Orthopaedic Surgery, New York University Medical Center, New York, NY 10003 USA; 2 Department of Rheumatology, The First Affiliated Hospital of Soochow University, Suzhou, Jiangsu 215006 PR China; 3 Department of Cell Biology, New York University School of Medicine, New York, NY 10016 USA

**Keywords:** Systemic lupus erythematosus, Progranulin, Inflammation, Anti-dsDNA antibody, Renal lesion

## Abstract

**Backgrounds:**

Progranulin (PGRN) is a growth factor-like molecule with diverse roles in homeostatic and pathogenic processes including the control of immune and inflammatory responses. Pathogenic inflammation is a hallmark of systemic lupus erythematosus (SLE) and elevated serum levels of PGRN has been evaluated as a biomarker of disease activity in SLE. However, the role of PGRN in SLE has not been fully investigated. This study is aimed to determine the potential involvements of PGRN in SLE.

**Methods:**

Wild type (WT) and PGRN knockout (PGRN^-/-^) C57BL/6 mice received intraperitoneal injection of pristane for induction of a murine model of SLE. Sera were collected every biweekly and levels of anti-dsDNA antibody, IgG, and inflammatory factors were measured. Mice were sacrificed 5 months later and the renal lesions, as well as the proportions of T cell subtypes in the spleen were analyzed.

**Results:**

Following exposure to pristane, PGRN^-/-^ mice generated significantly lower levels of anti-dsDNA antibody and IgG relative to WT mice. PGRN^-/-^ mouse kidneys had less IgG and collagen deposition compared with WT mice after pristane injection.

**Conclusion:**

The results indicate that PGRN participates in inflammatory response and renal damage in pristane induced SLE models, suggesting that PGRN mediates the onset of SLE.

## Introduction

Systemic lupus erythematosus (SLE) is an autoimmune disease affecting a large group of populations worldwide [1, 2]. This disease presents with marked clinical heterogeneity, impacting multiple organ systems with mild to extreme severity and non-linear inflammatory organ and tissue damage over a chronic course [3]. Though improved disease monitoring and treatment options have reduced overall mortality in SLE patients, treatment-resistant disease progression and symptoms together with detrimental effects of available immunosuppressive therapies highlight the need for expanded understanding of mechanisms driving SLE toward the development of early diagnostic methods and better treatment options [4]. This demands further investigations on suspicious SLE-prompting factors including progranulin (PGRN) [5].

Characterized by generation of autoantibodies and sustained immune response to nuclear and non-nucleic acid-associated autoantigens, SLE is resulted from loss of adaptive tolerance and the activation of the innate immune system. Expression of circulating inflammatory mediators can serve as serological markers of SLE conjunctive to autoantibody presentation. Such markers include elevated expression of cytokines such as IL-6, TNF-α, IL-10, IL-17, IL-23, and IFN-γ [6–9].

Serious SLE-associated renal complications, termed lupus nephritis (LN), are common and associated with poor prognosis and significantly reduced life expectancy [10, 11]. Recent findings elucidate an essential role of distinct subsets of T helper (Th) cells in driving LN phenotype. Th1 and Th17 cells have been implicated in the development of diffuse proliferative lupus nephritis, while Th2 cytokines drive development of membranous lupus nephritis. These data support the hypothesis that Th1/Th2 balance is one of the critical determinants for histopathology of LN [12].

Progranulin (PGRN), a cysteine-rich secreted growth factor-like molecule, has been identified in the search for markers of disease activity in SLE. PGRN is produced by a wide variety of tissues and cell types and has multiple functions in key processes implicated in disparate diseases and conditions including autoimmunity, insulin resistance, and neurodegeneration [13–16]. Serum levels of PGRN are significantly higher in SLE patients compared with non-affected individuals and the levels have been evaluated as a useful biomarker of disease activity and therapeutic response in SLE [17–19]. Moreover, PGRN is positively correlated with the level of anti-double-stranded DNA (anti-dsDNA) antibody, and negatively correlated with levels of complement components CH50, C3, and C4 [17]. Only a very few studies have examined the role and mechanism of PGRN in SLE. Using pristane induced murine lupus model, Chunmei Jing et al. showed that compared with WT SLE mice, inflammatory cell infiltration, tissue edema, and necrosis were alleviated in PGRN^-/-^ SLE mice [20]. Another team have found that overexpression of granulin (released from PGRN) in vivo exacerbated LN, whereas down-regulation of granulin ameliorated LN [21].

In this study, we employed a murine pristane-induced lupus model in wildtype (WT) and progranulin knockout (PGRN^-/-^) C57BL/6 background mice to better assess the role of progranulin in SLE.

## Materials and methods

### Mice

All animal studies were performed in accordance with institutional guidelines and under approval from the Institutional Animal Care and Use Committee of New York University. C57BL/6 mice were obtained from Jackson Laboratories. The generation and characterization of PGRN knockout (PGRN^*-/-*^) mice has been described previously [22]. Mice were housed within the rodent barrier facility at the Skirball Institute of Biomolecular Medicine with ad libitum access to food and water in a specific pathogen free room under controlled temperature and humidity on a 12-hour light/dark cycle.

### Generation of systemic lupus erythematosus model

Generation of the SLE model was conducted in accordance with previous reports [23]. 8-week-old female C57BL/6WT and PGRN^-/-^ mice were randomly divided into treatment groups (5 mice per group) for single intraperitoneal injection of 0.5 mL vehicle (sterile phosphate buffered saline; PBS) or pristane (P2870, Sigma-Aldrich, USA). Mice were subjected to retro-orbital bleeding at 2-week intervals until the end point of the experiment. 5 months after intraperitoneal injection, mice were sacrificed via CO_2_ overdose followed by cervical dislocation; kidneys and spleens were collected for further analysis.

### Histological analysis

For histological analysis, excised kidneys were immediately fixed in 4% paraformaldehyde followed by pre-processing and paraffin embedding. Serial 5μm-thick tissue sections were stained with Masson’s Trichrome according to the manufacturer’s instructions (HT15, Sigma-Aldrich, USA). For fluorescence staining of IgG, frozen sections were blocked with 5% normal goat serum (005-000-121, Jackson Immunoresearch Inc., USA) and then incubated with Alexa Fluor 488 conjugated goat anti-mouse IgG antibodies (A-11001, Sigma-Aldrich, USA) for 30 min. Pictures were acquired with Leica microscope (Leica) and evaluated using image-pro plus software.

### Detection of circulating IgG, anti-dsDNA antibodies and inflammatory cytokines

Levels of anti-dsDNA antibodies in serum were measured by ELISA assay as described previously [24, 25]. In brief, ELISA plates (9018, Corning, USA) were pretreated with 10ug/ml methylated BSA (A1009, Sigma-Aldrich, USA) at 37 °C for 1 hr and coated with 2.5ug/ml calf thymus dsDNA (D8515, Sigma-Aldrich, USA) overnight at 4 °C. Wells were blocked with 1% gelatin and washed twice with PBST prior to 2 hr incubation with mouse sera which was diluted in EDTA solution containing 0.1% gelatin. Following incubation, wells were aspirated and washed twice with PBST prior to development using horseradish peroxidase (HRP)-conjugated goat anti-mouse IgG antibody (1036-05, Southern Biotech, USA) and tetramethylbenzidine (TMB) substrate (S1601, Aligent, USA). Commercial ELISA kits were used according to the manufacturer’s instructions for assessment of total IgG (88-50400-88, Invitrogen, USA), IgG1 (88-50410-22, Invitrogen, USA), IgG2a (88-50420-22, Invitrogen, USA), TNFα (88-7324-77, Invitrogen, USA) and IL-6 (88-7064-88, Invitrogen, USA) levels. Absorbance at 450 nm was recorded using a plate reader (Spectramax MiniMax i3x, Molecular Devices, USA).

### Splenocyte isolation

Spleens were individually placed into a sterile petri dish and finely minced before passage through a 70 µm cell strainer. Dishes and strainers were rinsed with a total 10 mL of complete media comprised of DMEM (10-013-CM, Mediatech, USA) supplemented with 10% v/v fetal bovine serum (s11150, Atlanta Biologicals, USA) and penicillin-streptomycin (P4333, Sigma-Aldrich, USA). Cells were centrifuged at 800 g for 5 min. Supernatants were discarded and pellets were resuspended in 1 mL Ammonium-Chloride-Potassium lysis buffer (A1049201, Glibco, USA) and incubated for 5 min prior to two washes with complete DMEM and centrifugation at 800 g for 5 min. Cell viability and count were determined via Trypan Blue (T8154, Sigma-Aldrich, USA) staining and resuspended for FACS analysis.

### Intracellular FACS analysis

Freshly isolated spleen cells were incubated in culture medium containing phorbol-12-myristate-13-acetate (PMA; P8139, Sigma-Aldrich, USA; 10 nM),), ionomycin (I9657, Sigma-Aldrich, USA; 1 µM),) and brefeldin A (B5936, Sigma-Aldrich, USA; 10 µg/mL) for 6hrs, followed by FVS700 incubation for dead cell exclusion. Cells were fixed and permeabilized prior to staining for flow cytometry. Th1, Th2, Th17, and Treg cell populations were investigated via staining with anti-CD4-FITC (100405), anti-IFNγ-PerCP (505821), anti-IL4-PE (504103), anti-IL17-APC (506915), anti-CD25-PE (102007), and anti-Foxp3-Alexa Fluor 647 (126407) purchased from Biolegend Inc., USA. All data were analyzed using FlowJo software (version 9.9.5; BD Biosciences).

### Statistical analysis

Data are expressed as mean ± SD from biological replicate of 5 mice per group. Images provided are representative of independent experiments. Statistical analyses performed by two-way ANOVA or T test using the GraphPad Prism 8.0 program. A value of *p* < 0.05 was considered statistically significant.

## Results

### PGRN^-/-^ SLE mice generate lower levels of anti-dsDNA antibody and IgG

**Fig. 1:**
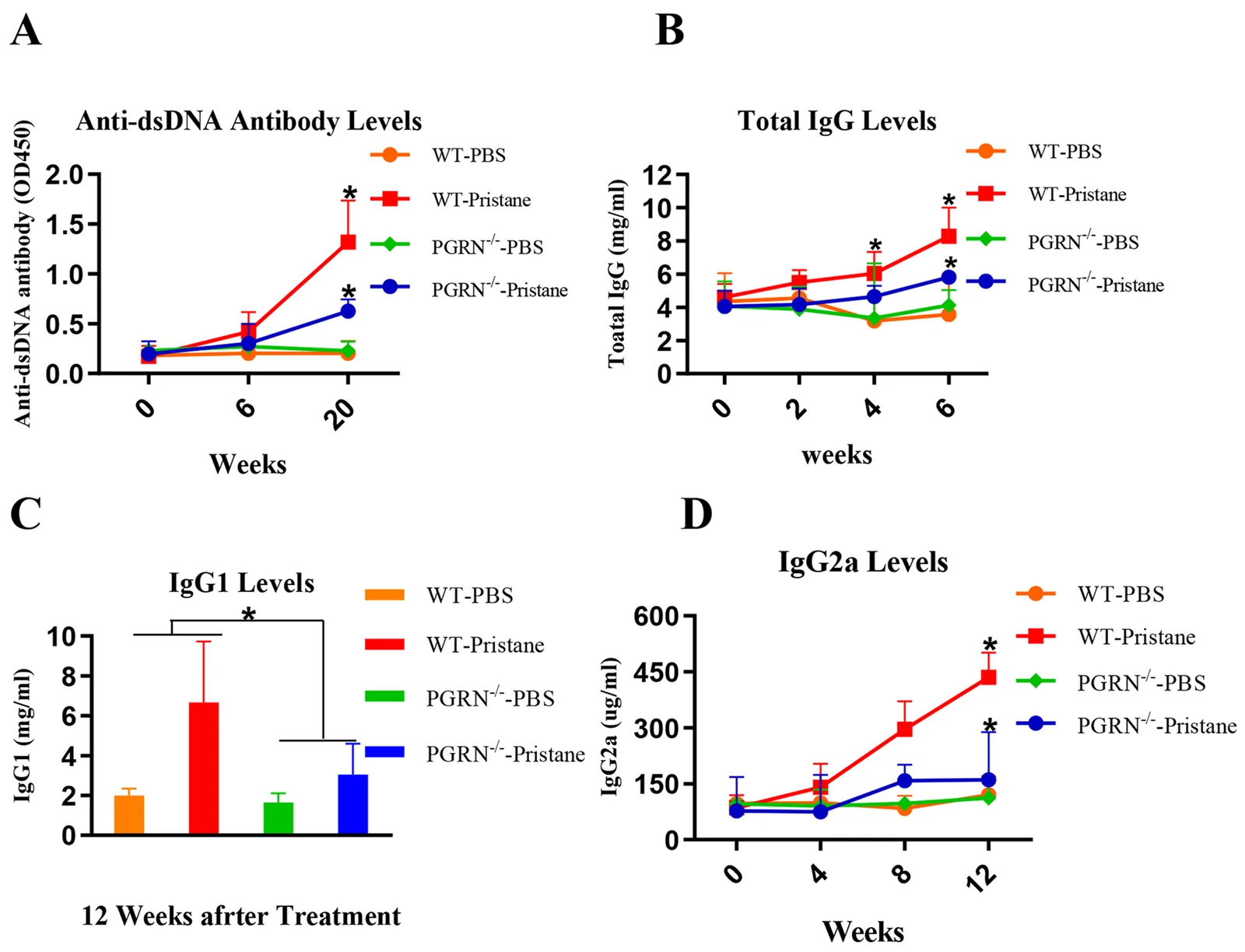
PGRN^-/-^ mice show lower levels of anti-dsDNA antibody and immunoglobulin compared with WT mice following administration of pristane. Sera were collected at different time points after pristane injection, anti-dsDNA antibody and immunoglobulin levels were determined by ELISA. **A** Anti-dsDNA antibody levels, **B** Total IgG levels, **C** IgG1 levels, **D** IgG2a levels. Results are the means ± SD of 5 mice per group. * *p* < 0.001

Pristane or PBS were injected into WT and PGRN^-/-^ female C57BL/6 mice and anti-dsDNA antibody as well as IgG levels were monitored. ELISA results showed that anti-dsDNA antibody levels in the PGRN^-/-^ group were almost the same as that in the WT group in the absence of pristane, however it began to increase in the pristane treated group at the 6th week after pristane injection. This trend persisted through to the experimental endpoint at 20 weeks after pristane administration, and levels of anti-dsDNA antibody had almost doubled in the WT-Pristane group at this timepoint compared with PGRN^-/-^ Pristane group (WT- PBS vs. WT-Pristane and WT- Pristane vs. PGRN^-/-^ Pristane, both *P* < 0.001; Fig. 1A). In the WT group, the levels of total IgG, IgG1, and IgG2a were all significantly elevated at indicated time points after pristane injection (*P* < 0.05; Fig. 1B–D). In the PGRN^-/-^ pristane group, total IgG increased obviously (*P* < 0.05) but this elevation did not reach levels observed in the WT group after treatment (we could not get enough sera for multiple tests so the following data were from different time points) (Fig. 1B and C).

### Renal lesion severity is attenuated in PGRN^-/-^ SLE mice

**Fig. 2:**
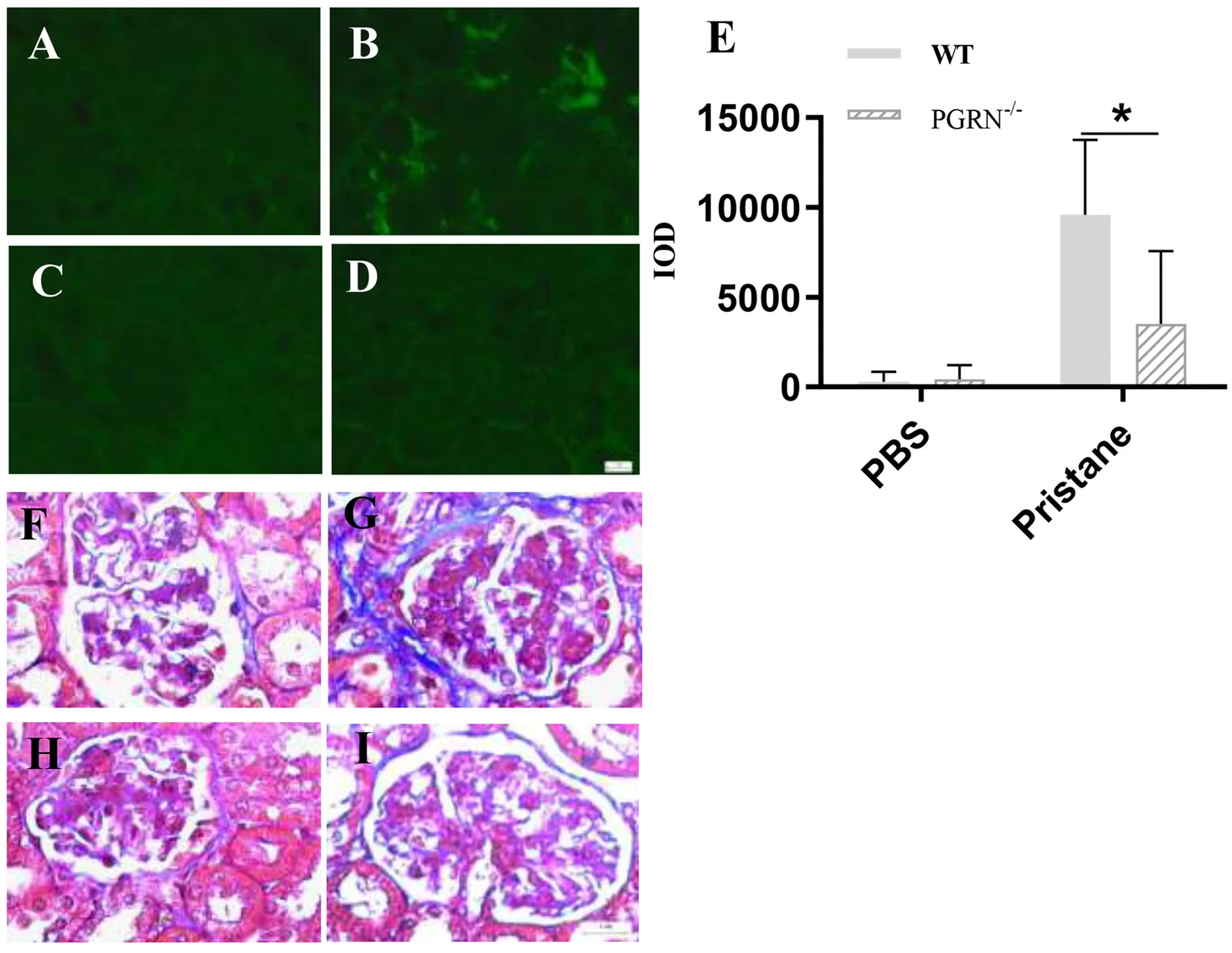
PGRN^-/-^ kidneys exhibit attenuated IgG and Collagen deposition following SLE-induction. Mouse kidneys were obtained at the end of experimental period, 5um thick sections were stained with Alexa Fluor 488 conjugated goat anti-mouse IgG as shown in **A**–**E. A** WT mice injected with PBS, **B** WT mice injected with pristane, **C** PGRN^-/-^ mice injected with PBS, **D** PGRN^-/-^ mice injected with pristane, **E** Integrated optical density (IOD) of the four groups. Masson’s Trichrome staining results are shown in **F**–**I. F** WT mice injected with PBS, **G** WT mice injected with pristane, **H** PGRN^-/-^ mice injected with PBS, **I** PGRN^-/-^ mice injected with pristane. Sections were observed by fluorescence or light microscope (400×) and quantified in image-pro plus. Data were obtained from five to six fields from 5 mice per group. *p < 0.05

Mouse kidneys were collected for analysis 5 months after injection with PBS or pristane. Kidneys were cut into 5 µm-thick serial tissue sections for histological and immunofluorescence staining. Immunofluorescence staining indicated no difference in IgG deposition attributable to genotype in naïve animals while pristane-treated PGRN^-/-^ mice exhibited reduced signal relative to the pristane-treated WT mice (Fig. 2A–E). Sections were also stained with Masson’s Trichrome for analysis of collagen deposition. While no obvious differences were observed with PBS treatment (Fig. 2F and H), PGRN^-/-^ mice exposed to pristane exhibited reduced collagen-positive areas (reflected by blue staining) relative to their WT counterparts (Fig. 2G and I).

### Th1 immune cell populations are altered in PGRN^−/−^ pristane mice

**Fig. 3:**
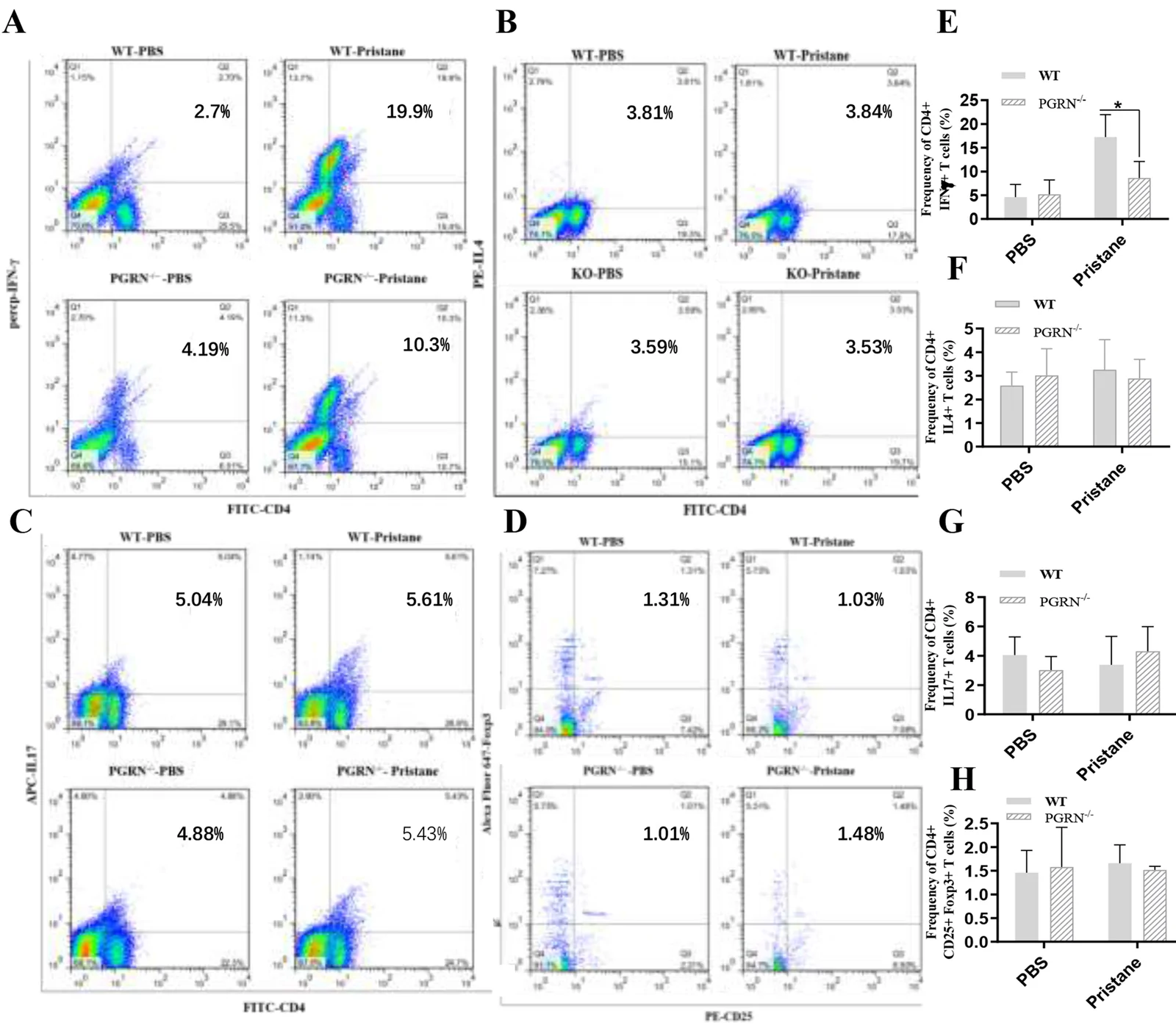
PGRN^−/−^ pristane mice exhibit altered T cell response. Mice were sacrificed and splenocytes were obtained 5 months following pristane administration. **A** FACS analysis of CD4+IFN-γ+T cells, **B** FACS analysis of CD4+IL4+T cells, **C** FACS analysis of CD4+IL17+T cells, **D** FACS analysis of CD4+CD25+Foxp3+T cells, **E** Percentage of CD4+IFNγ+T cells, **F** Percentage of CD4+IL4+T cells, **G** Percentage of CD4+IL17+T cells, **H** Percentage of CD4+CD25+Foxp3+T cells in the spleens of WT and PGRN^-/-^ mice. Values are means ± SD of 5 mice per group, * *p* < 0.05

T helper (Th) cells play an essential role in the pathogenesis of SLE. In this study, we obtained splenocytes from WT and PGRN^-/-^ mice 5 months after administration of pristane or vehicle and measured the percentage of Th1, Th2, Treg and Th17 cells by flow cytometry. Analysis revealed an increase in Th1 cell populations associated with pristane exposure, though PGRN^-/-^ pristane mice exhibited significantly less Th1 cell frequency relative to their WT counterparts (*P* < 0.05; Fig. 3A and E). With regard to Th2, Treg and Th17 cells, there were no significant differences between the groups (all *P* > 0.05; Fig. 3B–D and F–H).

### PGRN^-/-^ mice generate lower levels of pro-inflammatory factors compared with WT mice after pristane injection

**Fig. 4:**
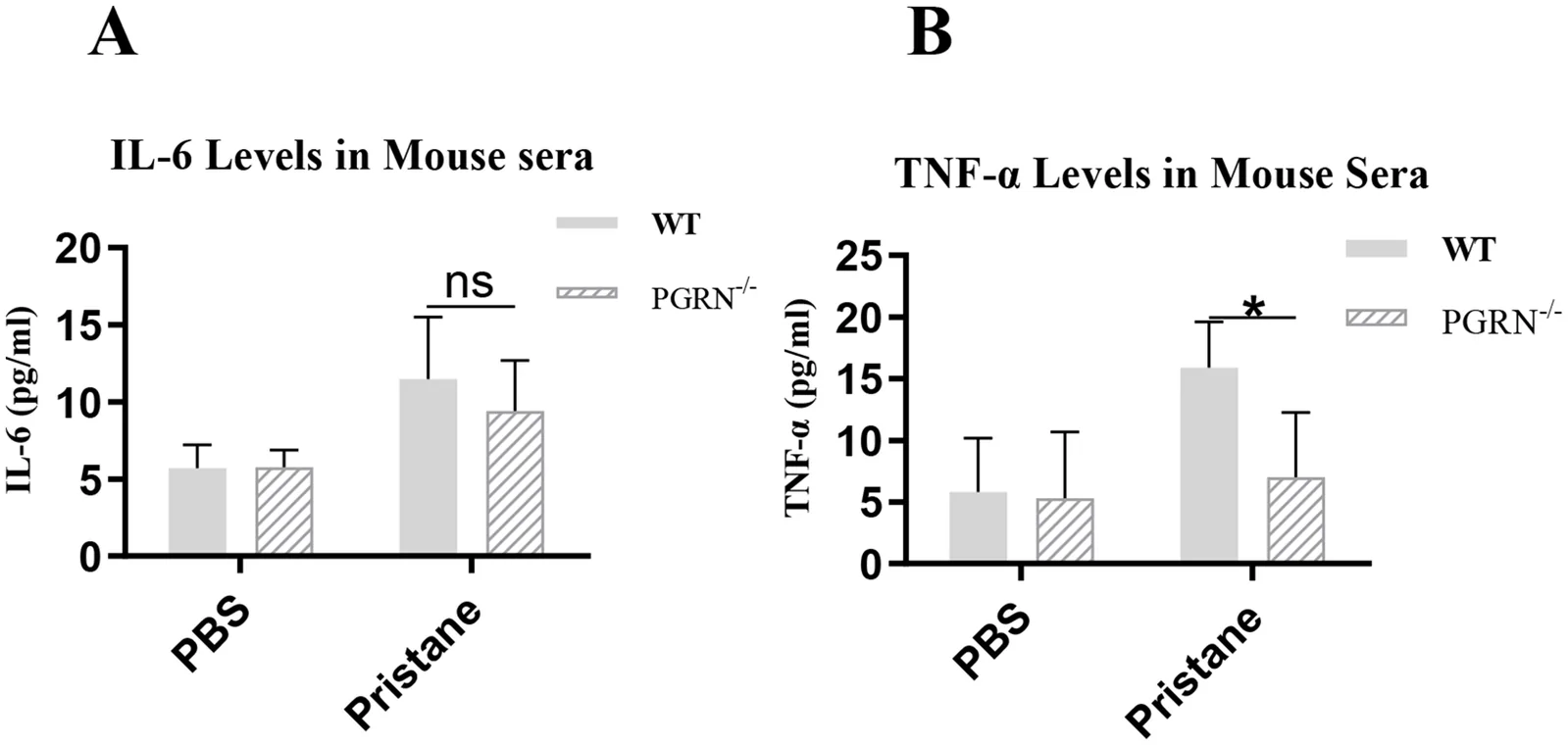
PGRN^-/-^ mice exhibit altered expression of circulating inflammatory factors following chronic pristane exposure. Mouse sera were collected 10 weeks after pristane injection, the levels of IL-6 (**A**) and TNF-α (**B**) were measured with commercial ELISA kits. Values are the mean concentrations ± SD from 5 mice per group. *p < 0.05

Ten weeks after pristane or PBS administration, levels of circulating IL-6 and TNF-ɑ were measured via ELISA. Results showed that IL-6 levels in the sera increased with pristane exposure in both WT and PGRN^-/-^ mice. PGRN^-/-^ -pristane mice, had lower serum levels of IL-6 relative to the corresponding WT group, but the difference did not reach statistical significance (*P* > 0.05; Fig. 4A). However, TNF-α levels in sera of PGRN^-/–^pristane mice were significantly lower than that of the WT-pristane group (*P* < 0.05; Fig. 4B).

## Discussion

In this study, we found that knockout of PGRN could attenuate serum concentrations of biomarkers of SLE such as anti-dsDNA antibody as well as the severity of pristane-induced SLE associated nephritis. Disease activity in lupus nephritis is often reflected in anti-dsDNA antibody levels; anti-DNA antibodies bind to nucleosomes and apoptotic cell debris to form immune complexes that are opsonized by complements which bind to the basal lamina of the kidney, causing chronic inflammatory and tissue damage [26–29]. Researchers have reported that levels of the multifunctional growth factor-like molecule PGRN are significantly elevated in SLE patients when compared to healthy controls, and there is a significant positive correlation between PGRN levels and anti-dsDNA and anti-ribosomal P0 antibodies [18, 30]. These findings suggest some connection between PGRN and classical markers and severity of SLE, however, they do not indicate a direct relationship between PGRN expression and autoantibody levels or tissue damage.

Histological processing of pristane-induced SLE mouse kidneys revealed reduced immunoglobulin and collagen deposition in the absence of PGRN in this study. Renal abnormalities are reflected in infiltration and aggregation of inflammatory mononuclear cells in the renal interstitium and deposition of immunoglobulin and collagen [31–33]. We also showed that serum levels of immunoglobulin subtype IgG and subclasses IgG1 and IgG2a were reduced in PGRN^-/-^ mice compared with WT mice after pristane injection. Upregulation of immunoglobulin, indicating activation of antibody-producing cells, is another common feature of SLE and contributes to development and progression of nephritis in accepted murine models of lupus [34, 35].

Many circulating cytokines are involved in SLE, though their relative contributions to disease activity and progression can be complex. In our model, TNF-α was significantly upregulated in the WT disease model while PGRN^-/-^ mice exhibited less change in expression of either cytokine following pristane exposure, and IL-6 increased mildly without significance in pristane induced SLE model of WT mice compared with PGRN^-/-^ mice. Master inflammatory mediator TNF-α is reported to have conflicting functions in this disease. Though a lot reports indicate that high levels of TNF-α is associated with SLE, a series of studies have proved that TNF-α blockers induced SLE specific autoantibodies as well as SLE-like symptoms [36]. A probable explanation is that TNF-α has a dual role in autoimmune diseases: on the one hand, it is associated with inflammatory activation within the dysregulated context of the SLE cytokine storm but on the other, it acts as an important inhibitory factor in SLE onset as having been shown to protect lupus prone mice from developing nephritis [37, 38]. Dr Shin also proposes that SLE could be caused by an increase in IL-17 levels after anti-TNF-α therapy [39]. As for IL-6, it is believed to correlate with disease activity and organ damage but not renal disorder [40].

The flow cytometry results in this study displayed there were more TH1 cells in the spleens of WT mice injected with pristane relative to the PGRN^-/-^ group. With regard to TH2, TH17 and Treg cell subsets, there were no differences among the four groups of mice. Different lymphocyte subsets participate in the onset of SLE and T cell balance is thought to play an important role in pathogenesis [8, 41–45]. Although SLE has been considered a Th2 cell driven condition [9, 46, 47], some studies have detected no subset predominance in peripheral Th1 or Th2 cells [41], while others suggest there is a TH1 bias in this disease [48, 49]. As for pristane induced SLE models, a strong predominance of Th1 has been reported [43, 50]. Recent findings have elucidated an essential role of Th1 and Th17 cells in the development of diffuse proliferative lupus nephritis, and Th2 cytokine in that of membranous lupus nephritis [8, 51, 52]. It has also been shown that, relative to healthy subjects, SLE patients exhibit significantly elevated levels of IL-17, IL-6, IL-12, and IL-10 [42]. At face value, our finding conflicts with some reports, this might because that the results were specific to the genetic status of mice and/or method of model induction [50].

PGRN has been shown to stimulate the propagation or activation state of various immunoregulatory cells including T cells and macrophages [21, 31, 32]. Recently, Katja Schmitz et al. reported that PGRN deficiency conferred resistance to autoimmune encephalomyelitis (EAE) in mice, PGRN deficiency contributed to MOG35-55-induced EAE resistance through prompting a reduction in MHC-II^+^ antigen presenting cells and consequent lessening of CD4+ and CD8+ cells alongside an increased scavenger cell population after immunization; conversely, PGRN mildly suppressed EAE progression when MOG35-55-immunization mediated generation of antigen-presenting cell populations in WT animals [33]. These results demonstrate the disease-, tissue-, cell-, and temporal specificity of PGRN’s multipotent functionality in mediating inflammation and immune response [53].

Our data confirm that PGRN level influences anti-dsDNA antibody titer in pristane-induced SLE models, as PGRN^-/-^ mice exhibit less severe nephritis than WT SLE mice, co-incident with attenuated upregulation of immunoglobulin and TNF-α which suggests that PGRN may directly or indirectly participate in the induction of several factors believed to regulate onset and progression of renal damage in SLE. To our knowledge, pristane-lupus is associated with abnormal production of IFN-a and IFN-ß, which have a central role in SLE [54]. Interferon-γ is required for lupus-like disease in MRL/lpr mice as well. However, progranulin has been reported to downregulate both IFN-a and TH1 cells [55, 56], this is contradictory to our data that PGRN^-/-^ mice generate less TH1 cells and show alleviated nephritis compared with WT mice after pristane challenge, which might be a result of complex cytokines and immune cells interaction involving both adaptive and innate immunity. Thus, the mechanism(s) and additional molecular mediators of PGRN’s influence in SLE warrants further investigation.

## Conclusion

PGRN participates in inflammatory response and renal damage in pristane induced SLE models as PGRN-/- mice generated significantly lower levels of anti-dsDNA antibody and had less IgG and collagen deposition in the kidney compared with WT mice after pristane injection. The main limitation of this research is lack of mechanism exploring, which should be demonstrated by more related upstream and downstream gene expression analysis using gene knockout /overexpression models.

## Data Availability

The datasets used and analyzed during the current study are available from the corresponding author on reasonable request.

## Abbreviations

PGRN: Progranulin
PGRN^-/-^ mice: PGRN knockout mice
Anti-dsDNA antibody: Anti-double-stranded DNA antibody
